# Nutritional Status and Anthropometric Indices in relation to Menstrual Disorders: A Cross-Sectional Study

**DOI:** 10.1155/2020/5980685

**Published:** 2020-11-23

**Authors:** Reihaneh Taheri, Fakhrodin Mesbah Ardekani, Hadi Raeisi Shahraki, Neda Heidarzadeh-Esfahani, Salimeh Hajiahmadi

**Affiliations:** ^1^Sepidan Bagherololoom Health Higher Education College, Shiraz University of Medical Sciences, Shiraz, Iran; ^2^Anatomy Department, Shiraz University of Medical Sciences, Zand Avenue, Shiraz 71348-45794, Iran; ^3^Department of Epidemiology and Biostatistics, School of Health, Shahrekord University of Medical Sciences, Shahrekord, Iran; ^4^Department of Nutrition, School of Public Health, Shahid Sadoughi University of Medical Siences, Yazd, Iran

## Abstract

**Purpose:**

Dietary habit and body composition can develop risk of menstrual disorders. The objective of this study was to assess the relationship between macronutrient intake, anthropometric indices, and menstrual disorders.

**Methods:**

This cross-sectional study was conducted on 217 women with an average age of 28.8 ± 7.9 years. Anthropometric indices including body mass index (BMI), waist circumference (WC), waist-to-hip ratio (WHR), and skinfold thickness from all participants were measured. Menstrual cycle characteristics were self-reported. The dietary habits were assessed by using a modified, semiquantitative 147 items Food Frequency Questionnaire (FFQ) by two trained dietitians. Chi-square and independent *T*-test were used to assess bivariate associations and logistic regression was implemented in SPSS 21.

**Results:**

Most of the participants (52.5%) suffered from at least one of the menstrual disorders including painful menstruation (41%), premenstrual syndrome (PMS) (24.9%), and irregular menstruation (22.1%). The mean of waist circumference in women with no complication was 76.0 ± 11.8 and in women with at least one disorder was 86.7 ± 14.0 (*P* < 0.001). Our results suggest that women with no disorder consumed less calorie, carbohydrate, protein, and fat in comparison to women with at least one disorder (*P* < 0.001). Furthermore, the proportion of all kinds of disorders among women, who had overweight or obesity, was significantly higher than women with normal BMI (*P* < 0.001).

**Conclusion:**

Irregular menstruation, painful menstruation, and PMS were significantly associated with high intake of calories, proteins, carbohydrates, and total fat. Furthermore, menstruation-related complications were worsened by obesity.

## 1. Introduction

Menstruation is a natural periodically uterine bleeding due to decreased production of estrogen and progesterone during healthy adolescent, premenopausal, and nonpregnant women's lives [1]. Menstrual cycle lasts average 3–7 days of bleeding with <80 ml of loss. There is a distance of 21–45 days between each cycle [2]. Women may experience menstrual disorders which affect their quality of lives and result in medical symptoms. Menstrual disorders are categorized according to the age of onset, duration, amount, and quality of bleeding [3]. Moreover, wide range of menstrual disorders included dysmenorrhea, premenstrual symptoms, oligomenorrhea, polymenorrhea, abnormal bleeding, amenorrhea, and menorrhagia [4] which can eventually lead to infertility [5]. The prevalence of menstrual disorders in Iran are as follows: dysmenorrhea (73.27%), oligomenorrhea (13.11%), polymenorrhea (9.94%), hypermenorrhea (12.94%), hypomenorrhea (5.95%), and menorrhagia (19.24%) [6]. Premenstrual syndrome (PMS) is a common disorder with prevalence of 54.9% among Iranian women [7]. It is characterized by irritability, confusion, mood swings, anxiety, social withdrawal, depression, and physical complaints such as bloating, breast tenderness or swelling (mastalgia), headache, and weight gain [8].

The lifestyle and dietary choice, mental condition, and physical activity have a strong influence on each woman who are prone to lifestyle-related diseases, such as menstrual disorders [9]. Dietary habit is one of the potential influencers on women's quality of life and health which can impact on several symptom of menstrual abnormality [8, 10]. Dietary habits such as eating breakfast and avoiding high-calorie density foods and junk foods (foods with high amount of salt, sugar, fat, or calories, and low nutrient content) positively associate with menstrual disorders. Dysmenorrhea is highly prevalent in girls who are eating fast food most of the time [9, 10]. Otherwise, Houghton et al. reported carbohydrate and fiber intake did not attribute to the risk of PMS [11]. On the other hand, the risk of dysmenorrhea was adversely associated with regular exercise and healthy physical activity in women [9]. There is a strong possibility that both overall and central obesity were significantly associated with having an irregular menstrual cycle [12]. Hip circumference and waist-to-weight and hip-to-waist ratio are other predictors/factors for menstrual disorders [13].

According to high prevalence of menstrual disorders [6], there are little consistent studies that examine both dietary intake and anthropometric indices with risk of menstrual disorders among Iranian women. The aim of this study is to determine the association between macronutrient intake and anthropometric indices with risk of common menstrual irregularity.

## 2. Materials and Methods

### 2.1. Study Design and Participants

This cross-sectional study was conducted on 217 females who were recruited from health centers in Sepidan city in Fars Province, Iran, from January 2018 to April 2018. Eligibility criteria for this study were Iranian women with 18–45 years of age who attended study clinics and started their menstruation more than 2 years ago and exclusion criteria were being currently pregnant or breastfeeding, consumption of hormonal contraceptives (including oral contraceptives, progestin-releasing intrauterine devices, and subdermal implanted contraceptives), use of intrauterine device (IUD), hysterectomy, and presence of type I or II diabetes, thyroid, or cardiovascular disorders. They were invited to answer the demographic questionnaire which consisted of age, education level, socioeconomic data, menstrual history, and marital status. Education level was categorized as school education, diploma, and university educations.

### 2.2. Ethics and Consent

The participants were completely informed about the situations of the study and accepted to enroll in this study with consent. Approval was granted from Shiraz University of Medical Sciences ethics committee before starting the study.

### 2.3. Assessment of Body Composition

Anthropometric measurements were taken at the health center by trained nutritionists and taken two times. Weight, height, body mass index (BMI), waist circumference (WC), wrist and thigh circumference at the right side, hip circumference, and waist-to-hip ratio (WHR) were measured. The height was measured with bare feet on the nearest 0.1 centimeter (cm) using a portable stadiometer with head in the Frankfort horizontal plane. Weight was measured with bare feet and light cloths on the nearest 0.01 kilogram (kg). Moreover, skinfold thickness at 4 sites of triceps, subscapular, hip, and suprailiac skinfolds at the right side was measured to the nearest 0.01 millimeter (mm) by a caliper. Each site was measured 3 times and the mean of the measures was reported as the final measure.

### 2.4. Assessment of Dietary Intake

The dietary habits were assessed by using modified, semiquantitative 147 items Food Frequency Questionnaire (FFQ) by two trained dietitians. The validity and reliability of FFQ were confirmed in the previous study in Iran [14]. The FFQ included a list of standard serving-sized foods which were commonly consumed by Persian population. Participants were asked to indicate their frequency of consumption (daily, weekly, monthly) of food items during the last year. Portion sizes of each food item were changed to grams by using household measures.

### 2.5. Assessment of Menstrual Cycle Features

In this study, we assessed three menstrual disorders including irregular menstruation, dysmenorrhea, and premenstrual syndrome. The menstrual disorders were determined by using a self-administered questionnaire and assessed using underlines criteria: (1) length of the menstrual cycle (normal cycle last 26–32 days), (2) duration of menses (3) amount of blood loss as reflected by the number of sanitary pads changed per day during menstruation. (4) Dysmenorrhea was assessed by asking them with or without severe pain during menstruation. (5) PMS was defined as one or more of the following symptoms starting 10 days before menstruation and disappearing at the start of period: rapid mood changes, depression, painful or tender breasts and bloating or swelling of the abdomen [15–17].

### 2.6. Statistical Analysis

Mean ± SD was reported for quantitative variables and number (%) for qualitative ones. To assess bivariate associations, chi-square and independent *T*-test were used. Finally, logistic regression was implemented in SPSS 21.0 and *P* < 0.05 set as statistically significant.

## 3. Results

A total of 217 healthy women with an average age of 28.8 ± 7.9 years were included in this study in which 61.7% of them were married. 113 out of 217 women (52.1%) had normal BMI (18.5–24.9 kg/m^2^), 63 (29.0%) were overweight (25–29.9 kg/m^2^), and 41 (18.9%) were categorized in the obese group (BMI ≥ 30).

**Figure 1:**
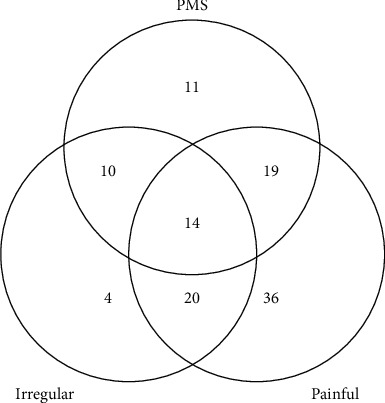
The number of women in each type of disorders.

Among 3 types of menstrual disorders studied, painful menstruation with 41% was the most prevalent menstrual disorder followed by PMS (24.9%) and irregular menstruation (22.1%). Moreover, most of the participants (52.5%) had at least one of the menstrual disorders (Figure 1).

Table 1 shows the demographic characteristics and Table 2 shows anthropometric indices and macronutrient intake of the participants with or without disorders. According to Iran's statistic center, economic status were divided into three groups including lower class (25.3%), middle class (40.6%), and high class (34.1%). Economic status had a significant relationship with having at least one of menstrual disorders (PMS, irregular, or painful menstruation) (Table 1).

In comparison to women with no menstrual disorder, women with at least one disorder had higher BMI, WC, energy, and carbohydrate, protein, and fat intake (*P* < 0.001). The mean ± SD of WC in women with no disorder was 76.0 ± 11.8 cm and in women with at least one disorder was 86.7 ± 14.0 cm (*P* < 0.001). Energy (kilocalorie) and fat (grams) intake of women with no disorder were 2260.5 ± 578.9 kilocalorie and 82.2 ± 27.5 grams (gr) while consumption of energy and fat in women with at least one disorder was higher, 2773.7 ± 804.7 calories and 104.8 ± 33.3 gr (Table 2).

Our results suggest that while there is no significant association between age and marital status with PMS or irregular or painful menstruation, a significant association between education level and PMS (*P* = 0.04) was seen (Table 3).

Furthermore, chi-square test showed that there was a significant association (*P* < 0.001) between BMI and all the menstrual disorders, as proportion of all kinds of disorders among women, who had overweight or obesity, was higher than women with normal BMI (Table 3). In all 3 types of disorders, women whom experienced the symptoms had significantly (*P* value < 0.05) higher WC, WHR, subcutaneous fat (subscapular, suprailiac, and triceps skinfolds) (Table 3) and energy, and carbohydrate and fat intake (Table 4).

The logistic analysis showed that considering other confounder factors, there was no significant association between understudy variables and menstrual disorders in women (Table 5).

## 4. Discussion

This study was conducted to determine the association between anthropometric indices, macronutrient intake, and menstrual abnormalities.

In this study, a significant relationship was observed between economic statuses with menstrual disorders (*P* value > 0.05) (Table 1). On the other hand, no significant relationship was reported between educational level and marital status with menstrual disorders (*P* value > 0.05). Although high educational level was associated with premenstrual syndrome (*P* value > 0.04), this association suggested that the higher level of education was probably associated with better information about healthy diet and lead to better comprehension of health status and menstrual symptom. Patsa et al. found no association between PMS with socioeconomic status (*P* value > 0.05) [16].

Our study showed that economic status has no significant effect on menstrual disorders even after being adjusted for potential confounders, including waist circumference, energy intake, carbohydrate intake, protein intake, body mass index, and fat intake (Table 5).

The anthropometric assessment revealed that 52.1% of participants had normal BMI, while 29.0% were overweight and 18.9% were obese. There were no underweight women found in this study. Analysis of our findings highlights the importance of the issue that dysmenorrhea, abnormal bleeding, irregular menstruation, and PMS were worsened by obesity (BMI > 24.9) significantly. Excess calorie intake can influence menstrual disorders. It was known that women affected by irregular menstrual cycles were overweight or obese [18]. The study of Carranza-Lira S et al. showed that BMI above 35 was associated with the greatest amount of bleeding [19]. Leptin (which is released from adipose tissue) has a role in the regulation of the gonadotropin during puberty, pregnancy, and lactation and considered explanation for menstrual abnormality in obese women. High circulating leptin level and leptin resistance seen in overweight women interact with the hypothalamo-pituitary-gonadal axis [20].

Testosterone, insulin, and sex hormone-binding globulin (SHBG) were attributed to increase odds of menstrual irregularity among obese women which are consistent with the literature suggesting that increased levels of insulin lead to decreased levels of SHBG [12] and associated with obesity both in central and peripheral adiposity [21].

There was a significant relationship between menstrual dysfunction and WC, wrist circumference, and WHR. Similarly, the study by Kafaei-Atrian et al. was conducted with the aim of association between the duration of menstrual bleeding and obesity-related anthropometric indices. It was shown that there was a significant statistic between menstrual duration and waist-to-height, waist-to-hip, hip-to-height, and arm-to-height ratios [13]. In the study by Rad et al., there was a significant correlation between dysmenorrhea and anthropometric indices such as height, WC, waist-to-height ratio, height-to- thigh ratio, WHR, and hip-to-thigh ratio [22]. Furthermore, subscapular skinfold, suprailiac skinfold, and triceps skinfold thicknesses (which are indicators of subcutaneous fat) were significantly attributed to menstrual disorders.

Our study revealed that women with all types of menstrual disorders had high daily macronutrient intake. Among previous studies, results have been inconsistent. Indriasari et al. evaluated the relationship between nutritional status, macronutrient intake, and menstrual disorders among 114 Indonesian adolescent girls and found that women who experienced menstrual disorders had inadequate daily macronutrient intake. There was no significant association of both carbohydrate and protein intake with menstrual disorder. It was found that many adolescents with adequate and excess carbohydrate and protein intake also experienced the menstrual disorder [23]. The previous study in a similar setting found a significant relationship between carbohydrate and menstrual disorders [24].

The present study has shown women with menstrual abnormalities consume a high fat diet. One animal study revealed that high fat diets induced obesity that starts cell cycle arrest and apoptosis of growth cycles. These have negative effects on ovarian functions [25]. The type of fatty acids concerned with menstrual disorders, for example, omega-3 fatty acids (e.g., fish and canola oil), reduced dysmenorrhea because of anti-inflammatory effects [17].

Some limitations should be taken into account when interpreting our results. First, due to the cross-sectional design of our study, the causality cannot be inferred. Second, dietary intake was assessed by FFQ that we could not deny measurement errors and misclassification of study participants. Third, menstruation characteristics were collected through self-reported questionnaires. Nevertheless, this questionnaire was asked by trained nutritionists.

## 5. Conclusion

In conclusion, a detailed investigation reflects most of the participants (52.5%) suffered from at least one of the menstrual disorders. It can be concluded that irregular menstruation, painful menstruation, and PMS were significantly associated with obesity and high intake of calories, proteins, carbohydrates, and total fat. However, considering other confounder factors, there was no significant association between understudy variables and menstrual disorders in this study.

## Figures and Tables

**Table 1:** Demographic characteristics of participants with or without menstrual disorders.

Characteristics		With no disorder (*n* = 103)	With at least one disorder (*n* = 114)	*P* value^*∗∗∗*^	Total (*n* = 217)
Age^*∗*^		28.1 ± 7.1	29.4 ± 8.5	0.20	28.8 ± 7.9
Marital status^*∗∗*^	Single	42 (50.0)	42 (50.0)	0.55	84 (38.7)
	Married	61 (45.9)	72 (54.1)		133 (61.3)
Education level^*∗∗*^	<dip	32 (39.0)	50 (61.0)	0.08	82 (37.8)
	Diploma	34 (47.9)	37 (52.1)		71 (32.7)
	>dip	37 (57.8)	27 (42.2)		64 (29.5)
Economic status^*∗∗*^	Low	23 (41.8)	32 (58.2)	**0.04**	55 (25.3)
	Middle	36 (40.9)	52 (59.1)		88 (40.6)
	High	44 (59.5)	30 (40.5)		74 (34.1)

^*∗*^Values reported as mean ± standard deviation. ^*∗∗*^Values reported as number (%). ^*∗∗∗*^Independent *t*-test (for age) and chi-square test were applied. *P* values in bold indicate numbers that are significant (*P* value < 0.05).

**Table 2:** The anthropometric indices and macronutrients intake in participants with or without menstrual disorders.

Characteristics		With no disorder (*n* = 103)	With at least one disorder (*n* = 114)	*P* value^*∗∗*^	Total (*n* = 217)
BMI (kg/m^2^)	Normal	74 (65.5)	39 (34.5)	**<0.001**	113 (52.1)
	Over weight	22 (34.9)	41 (65.1)		63 (29.0)
	Obese	7 (17.1)	34 (82.9)		41 (18.9)
Waist circumference (cm)^*∗*^		76.0 ± 11.8	86.7 ± 14.0	**<0.001**	81.6 ± 14.0
Energy intake (kcal)^*∗*^		2260.5 ± 578.9	2773.7 ± 804.7	**<0.001**	2530.1 ± 750.3
Carbohydrate intake (gr)^*∗*^		317.8 ± 82.4	389.2 ± 127.3	**<0.001**	355.3 ± 113.8
Protein intake (gr)^*∗*^		73.2 ± 16.6	82.7 ± 21.0	**<0.001**	78.2 ± 19.6
Fat intake (gr)^*∗*^		82.2 ± 27.5	104.8 ± 33.3	**<0.001**	94.1 ± 32.6

^*∗*^Values reported as mean ± standard deviation. ^*∗∗*^Chi-square test (BMI) and independent *t*-test were applied. *P* values in bold indicate numbers that are significant (*P* value < 0.05). BMI, body mass index.

**Table 3:** Various menstrual disorders across sociodemographic and anthropometric parameters.

Characteristics		Painful menstruation			PMS			Irregular menstruation		
		No	Yes	*P* value	No	Yes	*P* value	No	Yes	*P* value
Age^*∗∗*^		28.2 ± 7.2	29.7 ± 8.8	0.19	28.3 ± 7.5	30.3 ± 9.0	0.14	28.5 ± 7.6	29.7 ± 9.0	0.36
Marital status^*∗*^	Single	51 (60.7)	33 (39.3)	0.69	68 (81.0)	16 (19.0)	0.11	66 (78.6)	18 (21.4)	0.85
	Married	77 (57.9)	56 (42.1)		95 (71.4)	38 (28.6)		103 (77.4)	30 (22.6)	
Education level^*∗*^	<diploma	42 (51.2)	40 (48.8)	0.14	56 (68.3)	26 (31.7)	**0.04**	63 (76.8)	19 (23.2)	0.50
	Diploma	43 (60.6)	28 (39.4)		52 (73.2)	19 (26.8)		53 (74.6)	18 (25.4)	
	>diploma	43 (67.2)	21 (32.8)		55 (85.9)	9 (14.1)		53 (82.8)	11 (17.2)	
BMI^*∗*^(kg/m^2^)	Normal	80 (70.8)	33 (29.2)	**0.001**	96 (85.0)	17 (15.0)	**<0.001**	98 (86.7)	15 (13.3)	**0.001**
	Over weight	31(49.2)	32(50.8)		46(73.0)	17(27.0)		47(74.6)	16(25.4)	
	Obese	17(41.5)	24(58.5)			21(51.2)	20(48.8)		24(58.5)	17(41.5)
Waist circumference^*∗∗*^ (cm)		78.7 ± 13.5	85.9 ± 13.8	**<0.001**	79.5 ± 13.2	88.1 ± 14.5	**<0.001**	79.7 ± 13.5	88.3 ± 14.1	**<0.001**
Hip circumference^*∗∗*^ (mm)		51.9 ± 8.2	55.2 ± 8.6	**0.01**	52.3 ± 8.3	56.0 ± 8.8	**0.01**	52.3 ± 8.0	56.5 ± 9.4	**0.01**
WHR^*∗∗*^		0.77 ± 0.09	0.80 ± 0.06	**0.01**	0.77 ± 0.09	0.81 ± 0.06	**0.01**	0.78 ± 0.09	0.81 ± 0.05	**0.02**
Subscapular skinfolds^*∗∗*^ (mm)		22.7 ± 7.7	28.5 ± 11.9	**<0.001**	23.5 ± 8.4	29.9 ± 12.7	**<0.001**	23.8 ± 10.0	29.4 ± 8.8	**0.001**
Suprailiac skinfolds ^*∗∗*^(mm)		26.4 ± 9.0	30.5 ± 9.3	**0.001**	26.5 ± 8.8	33.0 ± 9.3	**<0.001**	26.8 ± 8.8	32.8 ± 9.6	**0.001**
Triceps skinfolds^*∗∗*^ (mm)		21.4 ± 6.2	24.6 ± 6.8	**<0.001**	21.3 ± 5.8	26.9 ± 7.2	**<0.001**	22.2 ± 6.8	24.6 ± 5.7	**0.03**

Values are reported as number (%) for descriptive and as mean ± standard deviation for quantitative variables. ^*∗*^Chi-square test and^*∗∗*^ independent *t*-test were applied. *P* values marked in bold indicate numbers that are significant (*P* value < 0.05). BMI, body mass index; WHR, waist-to-hip ratio.

**Table 4:** Various menstrual disorders across energy and macronutrient intake.

Characteristics	Painful menstruation			PMS			Irregular menstruation		
	No	Yes	*P* value	No	Yes	*P* value	No	Yes	*P* value
Energy intake (kcal)	2401.7 ± 678.7	2714.9 ± 811.5	**0.003**	2434.8 ± 720.8	2817.9 ± 770.8	**0.001**	2454.5 ± 698.6	2796.3 ± 866.1	**0.01**
Carbohydrate intake (gr)	339.6 ± 101.2	378.0 ± 127.1	**0.02**	341.0 ± 106.5	398.6 ± 124.9	**0.001**	343.2 ± 103.7	397.9 ± 136.9	**0.01**
Protein intake (gr)	75.8 ± 17.5	81.7 ± 21.8	**0.03**	76.3 ± 19.1	84.0 ± 20.0	**0.01**	77.4 ± 19.3	81.0 ± 20.3	0.27
Fat intake (gr)	87.5 ± 29.9	103.5 ± 34.3	**<0.001**	90.4 ± 32.5	105.3 ± 30.6	**0.003**	91.2 ± 32.0	104.2 ± 33.2	**0.02**

Values are reported as mean ± standard deviation. Independent *t*-test was applied. *P* values in bold indicate numbers that are significant (*P* value < 0.05).

**Table 5:** Logistic regression model showing the most associated factors on menstrual disorders.

Characteristics		OR	95% CI	*P* value
Waist circumference (cm)		1.02	0.97–1.07	0.37
Energy intake (kcal)		1.00	0.99–1.01	0.40
Carbohydrate intake (gr)		0.99	0.95–1.02	0.46
Protein intake (gr)		0.97	0.93–1.02	0.20
Fat intake (gr)		0.98	0.90–1.06	0.62
Economic status	Low	---		
	Middle	1.02	0.47–2.21	0.96
	High	0.50	0.23–1.09	0.08
BMI (kg/m^2^)	Normal	---		
	Over weight	1.86	0.64–5.38	0.25
	Obese	2.20	0.39–12.42	0.37

BMI, body mass index.

## Data Availability

The datasets used and analyzed during the present study are available from the corresponding author on reasonable request.

## Abbreviations

BMI: Body mass index
FFQ: Food Frequency Questionnaire
IUD: Intrauterine device
PMS: Premenstrual syndrome
WC: Waist circumference
WHR: Waist-to-hip ratio.
